# A Distributed Transmission Rate Adjustment Algorithm in Heterogeneous CSMA/CA Networks

**DOI:** 10.3390/s150407434

**Published:** 2015-03-26

**Authors:** Shuanglong Xie, Kay Soon Low, Erry Gunawan

**Affiliations:** School of Electrical and Electronic Engineering, Nanyang Technological University, 639798 Singapore; E-Mails: sxie2@e.ntu.edu.sg (S.X.); egunawan@ntu.edu.sg (E.G.)

**Keywords:** wireless sensor network, CSMA/CA, transmission rate adjustment, distributed processing

## Abstract

Distributed transmission rate tuning is important for a wide variety of IEEE 802.15.4 network applications such as industrial network control systems. Such systems often require each node to sustain certain throughput demand in order to guarantee the system performance. It is thus essential to determine a proper transmission rate that can meet the application requirement and compensate for network imperfections (e.g., packet loss). Such a tuning in a heterogeneous network is difficult due to the lack of modeling techniques that can deal with the heterogeneity of the network as well as the network traffic changes. In this paper, a distributed transmission rate tuning algorithm in a heterogeneous IEEE 802.15.4 CSMA/CA network is proposed. Each node uses the results of clear channel assessment (CCA) to estimate the busy channel probability. Then a mathematical framework is developed to estimate the on-going heterogeneous traffics using the busy channel probability at runtime. Finally a distributed algorithm is derived to tune the transmission rate of each node to accurately meet the throughput requirement. The algorithm does not require modifications on IEEE 802.15.4 MAC layer and it has been experimentally implemented and extensively tested using TelosB nodes with the TinyOS protocol stack. The results reveal that the algorithm is accurate and can satisfy the throughput demand. Compared with existing techniques, the algorithm is fully distributed and thus does not require any central coordination. With this property, it is able to adapt to traffic changes and re-adjust the transmission rate to the desired level, which cannot be achieved using the traditional modeling techniques.

## 1. Introduction

### 1.1. Motivation

IEEE 802.15.4 has become the *de facto* standard for wireless sensor networks [1] in a broad spectrum of applications such as home automation [2] and vehicle/satellite communications [3]. The flexibility of the carrier sensing multiple access with collision avoidance (CSMA/CA) enables the implementation of distributed networks with heterogeneous devices [4]. Many of these networks involve sensing and control tasks, which typically have lower traffics compared with normal wireless data network, but require each node to sustain certain throughput demand to guarantee system performance. For example, in a satellite attitude determination and control system (ADCS), various sensor nodes need to successfully transmit a number of packets containing sensor measurements to the controller within a certain time interval in order for the control signal to be properly computed and executed.

Subject to the throughput constraint, each node has the incentive to minimize its transmission attempts as more frequent transmission consumes more energy and occupies more channel resources. Thus, it is essential to tune each node’s transmission rate for both “private” and “public” benefits. This, in a homogeneous IEEE 802.15.4 CSMA/CA network, can be achieved using Markov modeling techniques [5,6,7,8], from which several key performance metrics such as packet success rate, packet delay can be derived and subsequently the optimal transmission rate can be carefully selected with respect to the given requirements.

However, in many wireless sensor network (WSN) and Internet of Things (IoT) applications, nodes are heterogeneous in that they have different throughput demands and thus different traffic loads. In addition, the traffic generated by each node may change over time. For example, a sensor monitoring a plant may refrain from sending its measurements to the controller unless the sensor readings are abnormal [9,10]. Consequently, a centralized Markov chain framework may not be flexible enough or may be too computationally expensive to be implemented online. Therefore, in order to fully exploit the flexibility of IEEE 802.15.4 protocol, it is strongly required to (a) devise a distributed mathematical framework to characterize the heterogeneous traffics and (b) to tune the transmission rate of each node to meet the specific performance requirements in a network with time-varying traffics.

### 1.2. Related Works and Contributions

A lot of efforts have been devoted to modeling the IEEE 802.15.4 CSMA protocol. Typically, a multi-dimension Markov chain approach [7,11] was employed to model the state evolution in a CSMA/CA network. From the model, the key performance metrics such as packet success rate, average delay, actual throughput [12] and energy consumption can be derived [13]. Such models are essential at the design phase as network designers can estimate the network performance simply by running the model and high accuracy can usually be expected. However, such models are complex and can thus only be applied in an offline manner. When frequent changes are expected, e.g., changes on number of active nodes, a Markov chain model can hardly serve to estimate the system performance at runtime. Furthermore, most of the models assume the homogeneity of the network as well as saturated traffics. Little attention has been paid to consider heterogeneous networks and unsaturated traffics that are very common in a variety of control and industrial applications [14,15,16]. The goal of this paper is thus to develop a distributed transmission rate tuning algorithm which is able to guarantee throughput requirement for IEEE 802.15.4 heterogeneous networks with varying traffics.

To implement such a tuning algorithm, it first requires the exploitation of the locally available information on MAC layer. Authors in [6] devised an approximation method to evaluate the performance metrics online using busy channel probability and transmission probability for a homogeneous CSMA/CA network. This work assumes that the number of nodes is known *a priori*, which is not the case for a time-varying network. The work in [17] explored the use of clear channel assessment (CCA) results for the tuning of binary exponent (BE) in order to achieve energy efficiency. In [18], authors used a frame-analytic mechanism to estimate the number of nodes and optimize the 802.15.4 parameters. The works in [19,20,21] investigated the pattern and optimal number of the clear channel assessments.

Furthermore, for transmission rate tuning, a distributed model or mathematical framework is also required to relate the local information to the estimated variables (e.g., the active number of nodes). This topic has been relatively less studied for IEEE 802.15.4 protocol compared with that of IEEE 802.11 protocol [22,23]. In [5], the authors proposed a dual-model approach to characterize the IEEE 8021.5.4 CSMA/CA protocol. Instead of using a multi-dimension Markov chain, the authors proposed two simpler Markov chains, namely node-state model and channel-state model. This simple construct is able to achieve comparable accuracy to complex modeling methods. Moreover, using the channel-state model as the baseline, we discovered in this paper that it is possible to devise a distributed algorithm that can estimate the network performance at runtime, not only for homogeneous networks, but also for heterogeneous networks.

In the context of distributed algorithms in CSMA/CA networks, quite a number of research works are focused on MAC layer parameter tuning [24]. The work in [8] used busy channel probability and transmission rate to minimize the energy consumption by tuning MAC layer parameters. A priority-based CSMA/CA was proposed in [25] to provide deadline-aware scheduling by varying the MAC layer parameters. Authors of [22] proposed an extended Kalman filter approach to estimate the number of active nodes in a homogeneous IEEE 802.11 network. The work in [23] proposed a sequential Monte Carlo (SMC) method for the same problem. The estimation results were then used to optimize the backoff parameters to achieve Nash equilibrium. The work in [26] uses the received power to determine the optimal transmit power and transmission rate for space capacity maximization. All these works, however, assume homogeneity of the network and the extension to heterogeneous networks is not straightforward.

In this paper, we consider the distributed rate adjustment for heterogeneous CSMA/CA networks. Compared with the aforementioned literature, the unique contributions of this work are summarized as follows:

- A mathematical characterization of heterogeneous traffics is provided, which enables each node to use CCA information to accurately determine the aggregate transmission rate of all other nodes. Compared with previous methods [8,22,23] that can only be applied for characterization and estimation in homogeneous networks, the proposed algorithm does not assume homogeneity of the network nor prior knowledge on nodes coexisting in the networks. It can thus be used for estimating both homogeneous and heterogeneous traffics;
- Based on the above mathematical framework, a distributed algorithm is proposed to predict the packet success rate and tune the transmission rate to meet the throughput demand. The algorithm is accurate with only an average error of 0.43% for homogeneous networks and 0.524% for heterogeneous networks.
- The algorithm is fully distributed and does not require any central coordination. Moreover, a change detection mechanism is also developed to allow each node to react promptly to on-going traffic changes and re-adjust its transmission rate to meet the throughput demand.

The rest of the paper is organized as follows. Section 2 reviews the CSMA/CA algorithm and discusses the model used throughout the paper. Section 3 derives the distributed algorithm, followed by the detectability analysis and the detection mechanism in Section 4. Extensive experimental results are presented in Section 5. Section 6 concludes the paper.

## 2. Preliminaries and Problem Formulation

### 2.1. IEEE 802.15.4 CSMA/CA Protocol

In this section, a brief overview of the IEEE 802.15.4 CSMA/CA protocol is first provided, focusing on the details that are related to the proposed study. A more comprehensive introduction can be found in [27].

The 802.15.4 is part of the IEEE family of standards that defines the physical layer (PHY) and medium access layer (MAC) for wireless personal area networks (WPAN). It is intended for devices with low power data rate, low complexity and stringent power requirement. The raw data rate in industrial, scientific and medical (ISM) band is 250 kbps. The basic time unit “slot” is defined as the unit backoff period which contains 10 bytes. In the time-slotted beacon-enabled mode, a superframe structure is used to govern the packet transmission. Each superframe begins with a beacon sent by the coordinator, followed by an active portion and an optional inactive portion. The active portion consists of a contention-access part and an optional contention-free part.

This paper considers the slotted CSMA/CA in the contention access portion. In a slotted CSMA/CA network, a node trying to transmit a packet would first initialize the counter called the number of backoffs (NB). Then the node performs the backoff algorithm to delay for a random number of time slots which is uniformly distributed in the range of $\left[\begin{array}{cc} 0, & 2^{BE}-1 \end{array}\right]$, where BE is the backoff exponent. When the backoff period is over, the node performs a clear channel assessment (CCA) to detect whether the channel is idle. The node would begin to transmit data if two consecutive CCAs are idle. Otherwise, the node would increase the number of NB and BE by one, without exceeding the maximum backoff limit *m* and maximum backoff exponent $m_{b}$ respectively. The packet will be discarded if NB exceeds *m*. Otherwise, the node will start to transmit a packet after performing two CCAs to confirm that the channel is idle. An ACK would indicate successful transmission. If the node fails to receive ACK due to collision or ACK timeout, the packet will be considered a failure assuming that no retransmission mechanism is implemented.

### 2.2. Problem Formulation

Consider a generic single-hop network where *N* nodes contend to transmit packets to a central node (*i.e.*, the coordinator) using the IEEE 802.15.4 slotted CSMA/CA protocol. Each node i is assigned a throughput demand of $1/t_{i}$. For convenience, $1/t_{i}$ is defined as the ratio of the number of successful packets to the number of time slots during a certain time period, *i.e.*, on average node *i* needs to successfully transmit a packet every $t_{i}$ slots. Due to the backoff failure and packet collision, packet transmissions may fail and the packet success rate of node i $p_{PSR_{i}}$ is smaller than 1. It is assumed in this paper that packet loss due to MAC layer contention dominates other forms of packet loss such as path loss and channel fading. This assumption is reasonable as for most applications that operate in close proximity, packet loss on physical layer is usually negligible [11,28].

Assume node i has a transmission rate of $1/{\text{λ}}_{i}$ (e.g., ${\text{λ}}_{i}=100$ means node i on average initialize a new packet transmission every 100 slots) and assume that $\lambda_{i}$ can be arbitrary tuned to meet the throughput demand. This feature applies to a wide variety of wireless industrial network systems [29,30,31]. For example, for network control systems employing data rate theorem [32], the transmission rate needs to be tuned frequently according to the channel condition to stabilize the control system. Another example would be a model-based network control system [31], where the sensor packet generation needs to be tuned according to the dynamics of the control system. $1/{\text{λ}}_{i}$ can also be viewed as a probability that node i initializes a new packet transmission in a random time slot. Further assume that each packet lasts for *L* slots and no retransmission is implemented on MAC layer (retransmission can be implemented on higher layer though). The objective of the proposed algorithm is for each node to find a suitable transmission rate $1/{\text{λ}}_{i}$ in a distributed manner such that:

(1) $$\frac{{p_{PSR}}_{i}}{\lambda_{i}}=\frac{1}{t_{i}}$$

Denote the vector $\text{λ}=\left({\text{λ}}_{1},{\text{λ}}_{2},\ldots ,{\text{λ}}_{N}\right)$ the optimal operating point where every node meets its throughput demand. It is assumed in this paper that such a point always exists, *i.e.*, the throughput demand is feasible. This assumption is reasonable in most sensor networks performing monitoring and control tasks as the data traffics generated by these nodes are usually below the capacity of the network.

**Figure 1:**
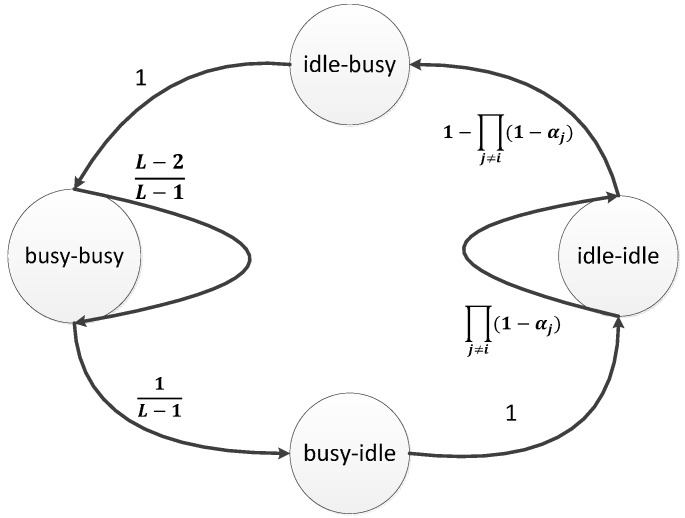
Channel state model.

In order to model the interactions in the network, it is essential to characterize the relationship between the two parameters of node *i*, namely the probability that node i starts to perform CCA in a random time slot ${\text{α}}_{i}$ and the probability that the CCA result is busy, namely busy channel probability ${\text{β}}_{i}$. Higher ${\text{β}}_{i}$ indicates that a node will on average perform more backoffs for a certain packet and thus higher ${\text{α}}_{i}$, and *vice versa*. This relationship can be quantified by calculating the average number of CCAs that node i experiences for each packet as follows:

(2) $${\text{α}}_{i}=\left(\overset{m-1}{\underset{k=0}{\sum }}\left(k+1\right)\left(1-{\text{β}}_{i}\right){{\text{β}}_{i}}^{k}+\left(m+1\right){{\text{β}}_{i}}^{m}\right)/{\text{λ}}_{i}=\frac{1-{{\text{β}}_{i}}^{m+1}}{\left(1-{\text{β}}_{i}\right)\lambda_{i}\;}$$

where $\sum_{k=0}^{m-1}\left(k+1\right)\left(1-{\text{β}}_{i}\right){{\text{β}}_{i}}^{k}+\left(m+1\right){{\text{β}}_{i}}^{m}$ enumerates all the possible number of CCAs and their corresponding probability. Furthermore, ${\text{β}}_{i}$ also depends on every other node’s probability of starting to perform CCA in a random time slot ${\text{α}}_{j}$. This relationship can be characterized by the channel state model in Figure 1 [5].

The idea of the channel state model lies in the property of the CSMA/CA that a node needs to sense two successive idle slots before transmitting a packet. The channel state model is thus constructed to figure out what is the probability that a random adjacent slot-pair is idle. As such, the slotted channel is modeled as a discrete Markov chain whose states, namely idle-idle, idle-busy, busy-idle, busy-busy, are on an adjacent slot-pair basis [5].

Each node i has a unique channel state model, which reflects the traffics of all other nodes. Its state evolves as follows. If none of other nodes perform any transmissions, the channel state will be idle-idle. This happens with the probability $\prod_{j\neq i}\left(1-\alpha_{j}\right)$. Otherwise, when at least one of the other nodes starts transmitting, the state goes to idle-busy and subsequently busy-busy. The state evolves to busy-idle once a packet transmission is completed, which happens with probability $\frac{1}{L-1}$. Otherwise, the state stays at busy-busy. Finally, once the state changes to busy-idle, it will go to idle-idle with probability 1.

From the state evolution, the stationary channel state can be solved as ${P_{II}}^{i}=\frac{1}{1+\tau_{i}\left(1+L\right)}$, ${P_{BB}}^{i}=\frac{\left(L-1\right)\tau_{i}}{1+\tau_{i}\left(1+L\right)}$ and ${P_{IB}}^{i}={P_{BI}}^{i}=\frac{\tau_{i}}{1+\tau_{i}\left(1+L\right)}$, where ${P_{II}}^{i}$, ${P_{BB}}^{i}$, ${P_{IB}}^{i}$ and ${P_{BI}}^{i}$ are the probabilities of node i that the slot-pair state is in idle-idle, busy-busy, idle-busy and busy-idle respectively, where $\tau_{i}=1-\prod_{j\neq i}\left(1-{\text{α}}_{j}\right)$.

The channel is considered busy when the slot-pair state is not ${P_{II}}^{i}$. Therefore, ${\text{β}}_{i}$ can be determined as:

(3) $${\text{β}}_{i}=1-{P_{II}}^{i}=\frac{\tau_{i}\left(1+L\right)}{1+\tau_{i}\left(1+L\right)}=\frac{\left(1-\prod_{j\neq i}\left(1-{\text{α}}_{j}\right)\right)\left(1+L\right)}{1+\left(1-\prod_{j\neq i}\left(1-{\text{α}}_{j}\right)\right)\left(1+L\right)}$$

The difference from the work in [5] is that instead of viewing the channel state as a whole, we model the channel from the perspective of an arbitrary node i. Each node has a unique channel state model. This slight yet important modification allows us to characterize the channel states in a distributed manner.

## 3. Distributed Transmission Rate Adjustment

Initially an arbitrary node *i*, with no prior information, tentatively transmits data at the rate ${\text{λ}}_{i}=t_{i}$. After a certain amount of time, node *i* can estimate the busy channel probability ${\text{β}}_{i}$ by recording the CCA information. The estimation is performed by calculating the ratio of the number of busy CCAs to the number of all the CCA for a certain period and using this ratio as the busy channel probability ${\text{β}}_{i}$. ${\text{β}}_{i}$ is then used to determine the optimal ${\text{λ}}_{i}$ [6,8]. From Equation (3), once ${\text{β}}_{i}$ is known, $\tau_{i}$ can be determined as:

(4) $$\tau_{i}=1-\underset{j\neq i}{\prod }\left(1-{\text{α}}_{j}\right)=\frac{{\text{β}}_{i}}{\left(1-{\text{β}}_{i}\right)\left(1+L\right)}$$

The term $\prod_{j\neq i}\left(1-{\text{α}}_{j}\right)$ contains transmission rate information of all other nodes j≠i and can be rewritten using Equation (2) as:

(5) $$\underset{j\neq i}{\prod }\left(1-{\text{α}}_{j}\right)=\underset{j\neq i}{\prod }\left(1-\frac{1-{{\text{β}}_{j}}^{m+1}}{\left(1-{\text{β}}_{j}\right)\lambda_{j}\;}\right)$$

One important observation is that for any 2 arbitrary node i and r, the backoff failure probability ${\text{β}}_{i}$ and $\beta_{r}$ are almost equal as long as the network size is not too small, or when the nodes are of homogeneous nature. This is because ${\text{β}}_{i}$ and ${\text{β}}_{r}$ are determined by $\prod_{j\neq i}\left(1-{\text{α}}_{j}\right)$ and $\prod_{j\neq r}\left(1-{\text{α}}_{j}\right)$ respectively and the ratio $\frac{\prod_{j\neq i}\left(1-{\text{α}}_{j}\right)}{\prod_{j\neq r}\left(1-{\text{α}}_{j}\right)}=\frac{\left(1-{\text{α}}_{r}\right)}{\left(1-{\text{α}}_{i}\right)}$ is very close to 1 as $\alpha_{r}$ and $\alpha_{i}$ are typically smaller than 0.01 [29,33]. Thus the subscript *i* for every ${\text{β}}_{i}$ can be dropped without affecting the analysis. Combining Equations (4) and (5), one can get:

(6) $$\text{β}=\frac{\left(1-\prod_{j\neq i}\left(1-\frac{1-{\text{β}}^{m+1}}{1-\text{β}}\;\frac{1}{{\text{λ}}_{j}}\right)\right)\left(1+L\right)}{1+\left(1-\prod_{j\neq i}\left(1-\frac{1-{\text{β}}^{m+1}}{1-\text{β}}\;\frac{1}{{\text{λ}}_{j}}\right)\right)\left(1+L\right)}$$

Our interest lies in finding a detailed characterization of the aggregate transmission rate $\sum_{j\neq i}\left(1/t_{j}\right)=\sum_{j\neq i}\left(1/{\text{λ}}_{j}\right)$ for ∀j≠i. However, it cannot be directly derived from Equation (6). Notice that in Equation (6), every $\frac{1-\beta^{m+1}}{1-\text{β}}\frac{1}{\lambda_{j}}$ is sufficiently small (typically far smaller than 0.01 for non-saturated applications [29,33]) and when expanding the polynomial $\left(1-\prod_{j\neq i}\left(1-\frac{1-{\text{β}}^{m+1}}{1-\text{β}}\frac{1}{\lambda_{j}}\right)\right)$, the second and higher order terms can be neglected without compromising the accuracy (e.g., the error when $\frac{1-\beta^{m+1}}{1-\beta }\frac{1}{\lambda_{j}}=0.01$ and *N* = 10 is less than 0.005). As such, Equation (6) can be rewritten in order to “isolate” ${\text{λ}}_{j}$ from other variables as follows:

(7) $$\text{β}\approx \frac{\frac{1-{\text{β}}^{m+1}}{1-\text{β}}\;\sum_{j\neq i}\left(1/\lambda_{j}\right)\left(1+L\right)}{1+\frac{1-{\text{β}}^{m+1}}{1-\text{β}}\;\sum_{j\neq i}(1/\lambda_{j})\left(1+L\right)}$$

From Equation (7), $\sum_{j\neq i}\left(1/t_{j}\right)=\sum_{j\neq i}\left(1/{\text{λ}}_{j}\right)$ can be given as:

(8) $$\underset{j\neq i}{\sum }\left(1/\lambda_{j}\right)=g\left(\beta \right)=\frac{\beta }{\left(1-\beta^{m+1}\right)\left(1+L\right)}$$

From Equation (8), the aggregate transmission rate is explicitly related to the busy channel probability. Once $\sum_{j\neq i}\left(1/t_{j}\right)$ is determined, estimation of the busy channel probability at the optimal operating point can be performed. In the following analysis, the superscript * is used to denote variables at the optimal operating point. The optimal transmission rate for node i can be expressed as $1/{{\text{λ}}_{i}}^{*}=\Delta^{*}/t_{i}$, where $\Delta^{*}$ is ratio of the optimal transmission rate $1/{{\text{λ}}_{i}}^{*}$ to $1/t_{i}$. According to the channel state model, when the transmission rate of node i is $\Delta^{*}/t_{i}$, the busy channel probability and the transmission rates of node j for ∀j≠i can be related as follows:

(9) $${\text{β}}^{*}=\frac{\left(1-\prod_{j\neq i}\left(1-\frac{1-{{\text{β}}^{*}}^{m+1}}{1-{\text{β}}^{*}}\;\frac{\Delta^{*}}{t_{j}}\right)\right)\left(1+L\right)}{1+\left(1-\prod_{j\neq i}\left(1-\frac{1-{{\text{β}}^{*}}^{m+1}}{1-{\text{β}}^{*}}\;\frac{\Delta^{*}}{t_{j}}\right)\right)\left(1+L\right)}$$

Using the same approximation technique as in Equation (7), Equation (9) can be rewritten as:

(10) $${\text{β}}^{*}=\frac{\frac{1-{{\text{β}}^{*}}^{m+1}}{1-{\text{β}}^{*}}\;\Delta^{*}\sum_{j\neq i}\left(1/t_{j}\right)\left(1+L\right)}{1+\frac{1-{{\text{β}}^{*}}^{m+1}}{1-{\text{β}}^{*}}\;\Delta^{*}\sum_{j\neq i}(1/t_{j})\left(1+L\right)}$$

Solving Equation (10) for $\Delta^{*}$ yields:

(11) $$\Delta^{*}=\frac{{\text{β}}^{*}}{\left(1-{{\text{β}}^{*}}^{m+1}\right)\;\sum_{j\neq i}\left(1/t_{j}\right)\left(1+L\right)}$$

In the meantime, $\Delta^{*}$ should satisfy $\Delta^{*}{p^{*}}_{PSR_{i}}=1$ according to Equation (1). The packet success rate ${p^{*}}_{PSR_{i}}$ of node *i* can be expressed as follows [34]:

(12) $${p^{*}}_{PSR_{i}}=\left(1-{{\text{β}}^{*}}^{m+1}\right)\underset{j\neq i}{\prod }\left(1-{\alpha^{*}}_{j}\right)$$

where $\left(1-{{\text{β}}^{*}}^{m+1}\right)$ is the probability that node i has access to an idle channel for a certain packet and $\prod_{j\neq i}\left(1-{{\text{α}}^{*}}_{j}\right)$ is the probability that at the same time there is no other node attempting to access the channel. Combining Equations (1), (7) and (12), one can obtain:

(13) $$\left(1-{\beta^{*}}^{m+1}\right)\left(1-\frac{1-{\beta^{*}}^{m+1}}{1-\beta^{*}}\Delta^{*}\underset{j\neq i}{\sum }\left(1/t_{j}\right)\right)=\frac{1}{\Delta^{*}}$$

The objective here is to estimate ${\text{β}}^{*}$ from $\sum_{j\neq i}\left(1/t_{j}\right)$. As such, one can substitute Equations (11)–(13) to eliminate $\Delta^{*}$ as follows:

(14) $$\underset{j\neq i}{\sum }\left(1/t_{j}\right)=f\left(\beta^{*}\right)=\frac{{\text{β}}^{*}-2{{\text{β}}^{*}}^{2}-{\beta^{*}}^{2}L+L{\text{β}}^{*}}{{\left(1+L\right)}^{2}\left(1-{\text{β}}^{*}\right)}$$

From Equation (14), it can be shown that $\sum_{j\neq i}\left(1/t_{j}\right)$ is monotonically increasing in ${\text{β}}^{*}$ when $0<{\text{β}}^{*}<\frac{L-{\left(L+2\right)}^{1/2}+2}{L+2}$ by differentiating $f\left({\text{β}}^{*}\right)$. Thus, an inverse function ${\text{β}}^{*}=f^{-1}\left(\sum_{j\neq i}\left(1/t_{j}\right)\right)=h\left(\sum_{j\neq i}\left(1/t_{j}\right)\right)$ exists in this region.

In conclusion, given the values of L, $\sum_{j\neq i}\left(1/t_{j}\right)$ and m, a unique ${\text{β}}^{*}$ can be readily solved and so is $\Delta^{*}$ (using Equation (11)). Node i can then use ${{\text{λ}}_{i}}^{*}=t_{i}/\Delta^{*}$ to determine its optimal transmission rate with respect to its throughput demand. In the actual implementation, the function ${\text{β}}^{*}=h\left(\sum_{j\neq i}\left(1/t_{j}\right)\right)$ can be discretized and computed using numerical methods and stored in a look-up table beforehand to simplify the computation.

## 4. Change Detection Mechanism

In this section, the analysis in Section 3 is extended to cases where there are new nodes joining the network or existing nodes leaving from the network during the operation. A detailed analysis is first provided on whether other nodes in the network can detect such changes. Then the problem of how each node can respond to such changes and adjust the transmission rate is addressed.

### 4.1. Detectability Analysis

**Figure 2:**
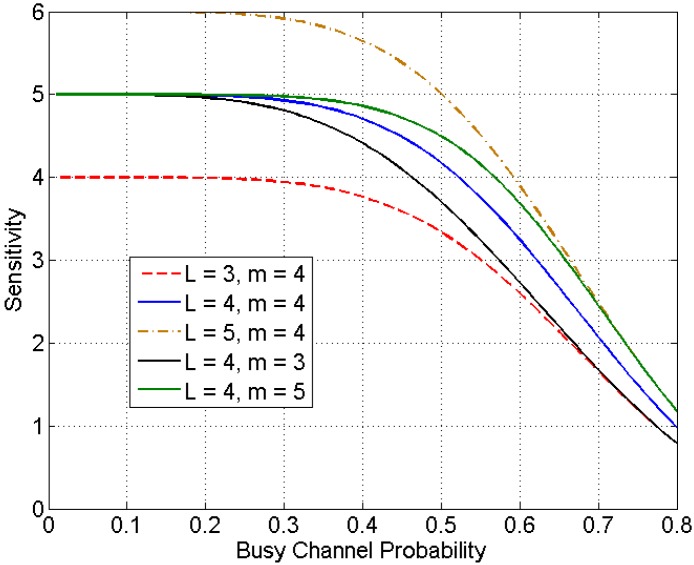
Sensitivity with respect to busy channel probability.

An arbitrary node i can detect such changes when there is a significant increase or decrease on β. The sensitivity of this detection mechanism is first analyzed. Any change on the number of nodes will first affect the value of $\sum_{j\neq i}\left(1/\lambda_{i}\right)$. The sensitivity of β in terms of $\sum_{j\neq i}\left(1/{\text{λ}}_{i}\right)$ can be derived by finding the derivative of $\text{β}=g^{-1}\left(\sum_{j\neq i}\left(1/t_{j}\right)\right)$. Since the expression of $g^{-1}\left(\sum_{j\neq i}\left(1/t_{j}\right)\right)$ can hardly be explicitly found, its inverse function g(β) is instead analyzed. It can be shown that its derivative $g^{\prime }\left(\text{β}\right)=\frac{1+m\beta^{m+1}}{{\left(1-\beta^{m+1}\right)}^{2}}\frac{1}{1+L}$ is a monotonically decreasing function and when β is small, $g^{\prime }\left(\text{β}\right)$ can be approximated as $g^{\prime }\left(\text{β}\right)\approx \frac{1}{1+L}$. According to the property of the derivative of inverse function, the derivative of $g^{-1}\left(\sum_{j\neq i}\left(1/{\text{λ}}_{i}\right)\right)$ is approximately (1+L) when β is small. When the busy channel probability is high, the slope of $g^{-1}\left(\sum_{j\neq i}\left(1/\lambda_{i}\right)\right)$ will decrease significantly. Figure 2 shows the sensitivity of β *versus* the busy channel probability for different combinations of m and L. It can be shown that when β<0.4, the sensitivity is close to 1+L. It drops quickly when the β>0.5 and the sensitivity is close to 1 when β=0.8. Note that for control and monitoring applications, the traffic load is relatively low and β rarely exceeds 0.5. Thus, every unit of change in $\sum_{j\neq i}\left(1/t_{j}\right)$ will in general be amplified to around 1+L units of change in β.

Furthermore, β is obtained by collecting the CCA information and is also subject to randomness. The randomness may also result in fluctuations in β even when there is no change on the number of active nodes. Thus, it is necessary to quantify such fluctuations and evaluate its impact on the proposed detection mechanism.

Each CCA can be considered as an independently identically distributed (*i.i.d*) process with a mean busy channel probability $\bar{\text{β}}$. In other words, each CCA is a Bernoulli trial and when T CCAs are performed for a single update interval, the number of busy results follows binomial distribution $B\left(T,\bar{\;\beta }\right)$. Furthermore, when $T\bar{\text{β}}>5$ and $\left(1-\bar{\text{β}}\right)>5$, the binomial distribution can be approximated as the normal distribution $N\left(T\bar{\text{β}},T\bar{\text{β}}\left(1-\bar{\text{β}}\right)\right)$ [35]. Thus, β also follows the normal distribution $N\left(\bar{\text{β}},\;\frac{\bar{\text{β}}\left(1-\bar{\text{β}}\right)}{T}\right)$. Using a α% confidence level, the fluctuation on β can be characterized by $\left[\begin{array}{cc} \bar{\text{β}}-\epsilon \sigma & \bar{\text{β}}+\epsilon \sigma \end{array}\;\right]$, where ϵ is the z-value corresponding to α% and $\text{σ}=\sqrt{\frac{\bar{\text{β}}\left(1-\bar{\text{β}}\right)}{\text{T}}}$.

Combining the results of the sensitivity analysis and the fluctuation analysis, it can be concluded that the changes on the traffic, namely $\sum_{l\in change}\left(1/t_{l}\right)$, can be detected when the change on β caused by the traffic exceeds the maximal fluctuation on β. In other words, $\sum_{l\in change}\left(1/t_{l}\right)$ can be detected with α% confidence if:

(15) $$\underset{l\in change}{\sum }\left(1/t_{l}\right)\geq \frac{2\epsilon \sqrt{\frac{\bar{\text{β}}\left(1-\bar{\text{β}}\right)}{T}}\left(1+m{\bar{\text{β}}}^{m+1}\right)}{{\left(1-{\bar{\text{β}}}^{m+1}\right)}^{2}\left(1+L\right)}$$

In actual implementations, as $\bar{\text{β}}$ is unavailable, the measured β can be used to approximate $\bar{\text{β}}$. It is also more convenient to use the update interval I (sec) instead of T. They can be easily related as $I=\frac{T\left(1-\bar{\text{β}}\right)\lambda_{i}}{\left(1-{\bar{\text{β}}}^{m+1}\right)RB_{slot}}$, where R is the data rate (bps) at PHY layer and $B_{slot}$ is the number of bits in a slot. Thus, Equation (15) can be rewritten as:

(16) $$\underset{l\in change}{\sum }\left(1/t_{l}\right)\geq \frac{2\epsilon \sqrt{\frac{\bar{\text{β}}{\left(1-\bar{\text{β}}\right)}^{2}{\text{λ}}_{i}}{IRB_{slot}}}\left(1+m{\bar{\text{β}}}^{m+1}\right)}{{\left(1-{\bar{\text{β}}}^{m+1}\right)}^{5/2}\left(1+L\right)}$$

**Figure 3:**
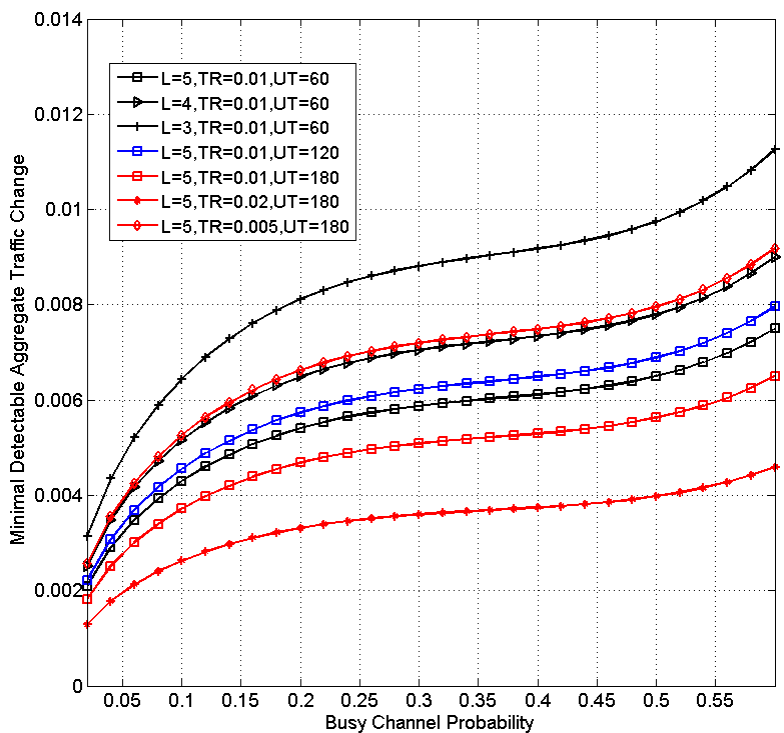
Minimal detectable aggregate traffic change *versus* busy channel probability (TR: Transmission rate; UT: Update interval (s)).

As an illustrative example, Figure 3 shows the minimal detectable traffic changes for different system scenarios for a typical IEEE 802.15.4-compliant node with data rate of 250 kbps and time slot length of 80 bits. A 95% confidence level is used.

It can be shown that as β increases, the proposed detection mechanism becomes less sensitive to traffic changes. It can also be shown from Equation (15) as well as Figure 3 that more sensitive detection can be obtained when (1) the packet length is longer; (2) when the transmission rate is higher or (3) when the update interval is longer.

Note that the minimal detectable aggregate traffic is also the upper bound of the error of traffic estimation. In actual implementations, as the errors are unbiased, the errors among different update intervals can be canceled and in the long run, the achievability of the throughput demand will not be compromised, as shown in Section 5.

### 4.2. Change Detection

When the busy channel probability is increased from ${{\text{β}}^{*}}_{1}$ to some higher detectable value, say ${\text{β}}_{2}$, there are new nodes joining the network. Any existing nodes i can then use Equation (4) to determine $\tau_{i}$. With Equation (5) and the approximation in Equation (7), we have:

(17) $$\tau_{i}=\overset{m}{\underset{k=0}{\sum }}{{\text{β}}_{2}}^{k}\left({\Delta^{*}}_{1}\sum_{j=1}^{N-1}\left(1/t_{j}\right)+\underset{l\in change}{\sum }\left(1/t_{l}\right)\right)$$

where node j∈(1,2,…,N−1) are all the existing node and node l∈change are the new nodes. As node i knows the values of ${\text{β}}_{2}$, $\tau_{i}$, $\sum_{j=1}^{N-1}\left(1/t_{j}\right)$ and ${\Delta^{*}}_{1}$, it can use Equation (17) to determine $\sum_{l\in change}\left(1/t_{l}\right)$ and then use Equations (11) and (14) to find the new optimal operating point. As for the new nodes l, a similar procedure can be performed to find the sum $\sum_{j=1}^{N-1}\left(1/t_{j}\right)$ but they first need to sense the ${\beta^{*}}_{1}$ for a short period before starting to transmit packets.

When the busy channel probability is decreased from ${{\text{β}}^{*}}_{1}$ to some lower value, say ${\text{β}}_{3}$, there are nodes leaving the network (*i.e.*, nodes become inactive), $\tau_{i}$ can be determined as:

(18) $$\tau_{i}=\overset{m}{\underset{k=0}{\sum }}{{\text{β}}_{3}}^{k}\left({\Delta^{*}}_{1}\underset{l\in current}{\sum }\left(1/t_{l}\right)\right)$$

where l∈current are the nodes still staying in the network. Using Equation (18), node i can readily determine $\sum_{l\in current}\left(1/t_{l}\right)$ and find the new operating point. The complete algorithm is summarized in Algorithm 1.

**Algorithm 1** Pseudo-code of the proposed algorithmc = 0; **for** k = 1; k < I; k++ **while** ($\text{c}++<c_{MAX}$)  Perform data transmission and record $\beta_{k}$ **end** **if** (k == 1)  $\sum_{j\neq i}\left(1/t_{j}\right)=g\left(\beta_{k}\right)$  $\lambda_{i}=$RateUpdate($\sum_{j\neq i}\left(1/t_{j}\right)$) **else if** ($\left|\beta_{k}-\beta_{last}\right|<\beta_{THR}$)  // Do not change the transmission rate **else if**
$\left(\beta_{k}>\beta_{last}\right)$  $\sum_{j\neq i}\left(1/\lambda_{j}\right)=g\left(\beta_{k}\right)$  Obtain new nodes’ traffic:  $\sum_{l\in change}\left(1/t_{l}\right)=\sum_{j\neq i}\left(1/\lambda_{j}\right)-\Delta^{*}\sum_{j\neq i}\left(1/t_{j}\right)$  $\lambda_{i}=$RateUpdate($\sum_{j\neq i}\left(1/t_{j}\right)+\sum_{l\in change}\left(1/t_{l}\right)$) **else if**
$\left(\beta_{k}<\beta_{last}\right)$  $\sum_{l\in current}\left(1/\lambda_{j}\right)=g\left(\beta_{k}\right)$  Obtain the current traffic:  $\sum_{l\in current}\left(1/t_{l}\right)=\sum_{l\in current}\left(1/\lambda_{j}\right)/\Delta^{*}$  $\lambda_{i}=$RateUpdate($\sum_{l\in current}\left(1/t_{l}\right)$) **end** c = 0**end****function**
$\lambda_{i}=$RateUpdate(Traffic) $\beta_{last}=\beta^{*}=h\left(\text{Traffic}\right)$ $\Delta^{*}=\Delta^{*}\left(\beta^{*},\text{Traffic}\right)$ (using Equation (11)) $\lambda_{i}=t_{i}/\Delta^{*}$**end**

In the table, k is the index of update interval and c is the time slot counter within an update interval. The change detection mechanism is triggered when the difference of busy channel probability of the current update interval ${\text{β}}_{k}$ and the last-triggered update interval ${\text{β}}_{last}$ exceeds some threshold $\beta_{THR}$.

## 5. Experimental Results

The proposed distributed algorithm has been implemented and extensively tested using the TelosB node [36]. TelosB consists of a Texas Instrument MSP430F1611 microcontroller and an IEEE 802.15.4-complaint CC2420 radio transceiver. The proposed algorithm is built on top of the TinyOS operating system [37], using a dialect of C language called nesC (network embedded system C language) [38]. The codes for the proposed algorithm occupy about 8-kB program memory. For the medium access control (MAC) layer and the interfaces to the radio stack, the IEEE 802.15.4 slotted CSMA/CA implementation [39] is used. The network is organized using star topology where multiple nodes transmit data to a pre-defined receiver. A packet transmission cycle consisting of transmitting the data packet and waiting and receiving the ACK packet is of 100 symbols long, which is translated to five time slots in the model in Section 2. The function value $h\left(\sum_{j\neq i}\left(1/t_{j}\right)\right)$ is discretized for $\sum_{j\neq i}\left(1/t_{j}\right)\in \left[0,\;0.1\right]$ with an interval of 0.0025 using a “float” type C array as a look-up table.

**Figure 4:**
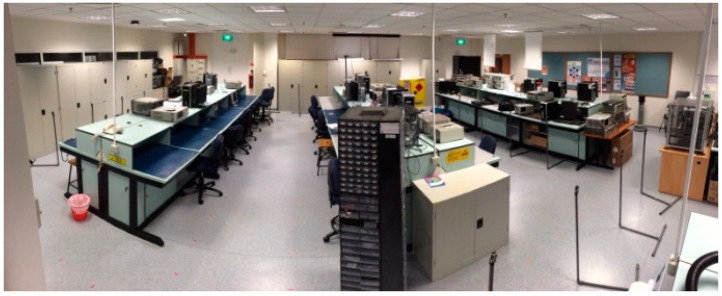
The testing area.

**Figure 5:**
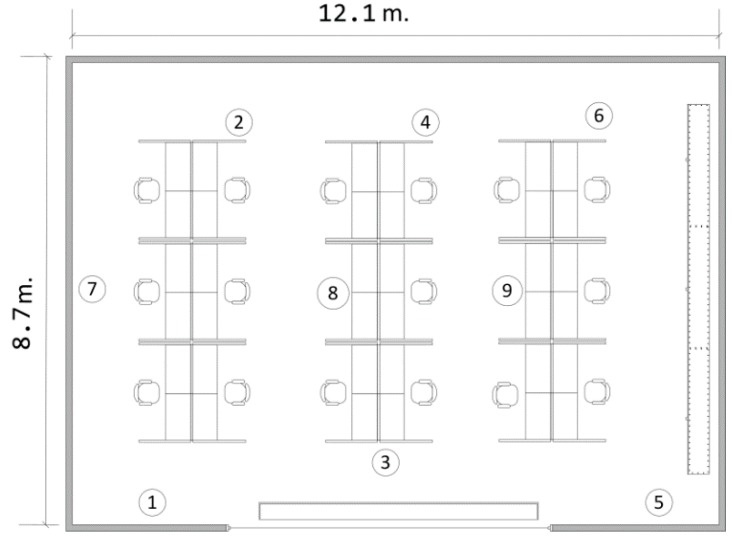
The floor plan of the testing area.

In the following discussions, the performance of the algorithm is evaluated under a typical laboratory environment, as shown in Figure 4 and Figure 5. Each node is placed on top of a 1-m PVC stand. The network to be tested is assumed to be a sparse and low-traffic network. The transmission power is set to 0 dBm. The application considered is an indoor laboratory monitoring and control system, where sensors of heterogeneous nature transmit packets to a central coordinator (controller). The traffic in such a system is unsaturated, but within a certain time interval, a minimum number of successful packets are expected (as “throughput demand”) for each sensor node in order to stabilize a control system, as discussed in [30,40], or to enable real-time monitoring. To take into account the effect of multi-path fading, the sensor nodes and the central coordinator are randomly placed in the nine spots in Figure 5 and are shuffled after each experiment.

The homogeneous case where each sensor node in the network has the same throughput demand is first tested. Different numbers of sensor nodes in the network are evaluated, ranging from three to seven. Each node is assigned a throughput demand of 1/200. Each experiment is run for 300 s and repeated 3 times and more than 5000 packets are transmitted by each node for each experiment.

**Figure 6:**
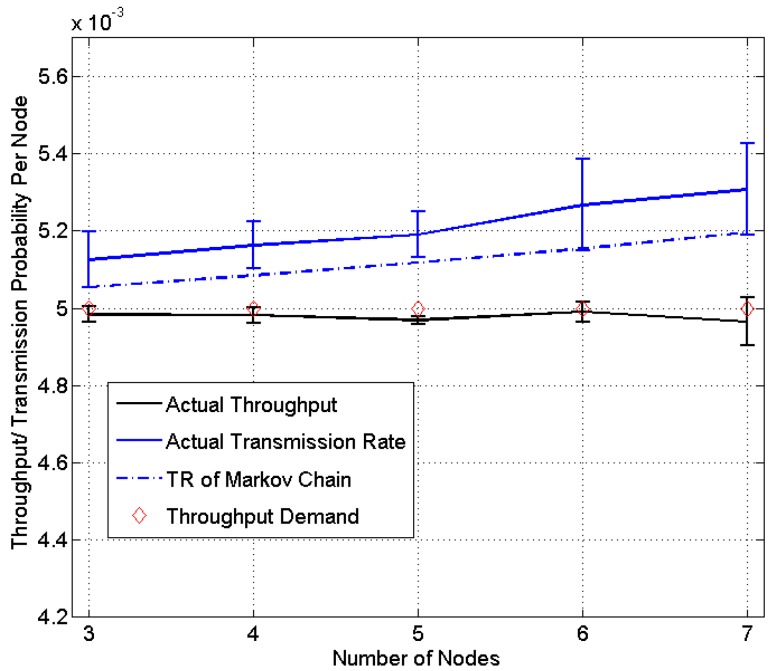
Experiment results of homogeneous networks (TR: transmission rate).

The result is shown in Figure 6. Figure 6 first compares the actual throughput (mean and standard deviation) *versus* the throughput demand. It is observed that the actual throughput is very close to the demand, with an average relative error of 0.43%. The transmission rate (mean and standard deviation) is also compared with the one predicted using the Markov chain model [5]. The result is in general very close but the proposed distributed algorithm performs slightly better in providing the optimal transmission rate based on the actual throughput.

**Figure 7:**
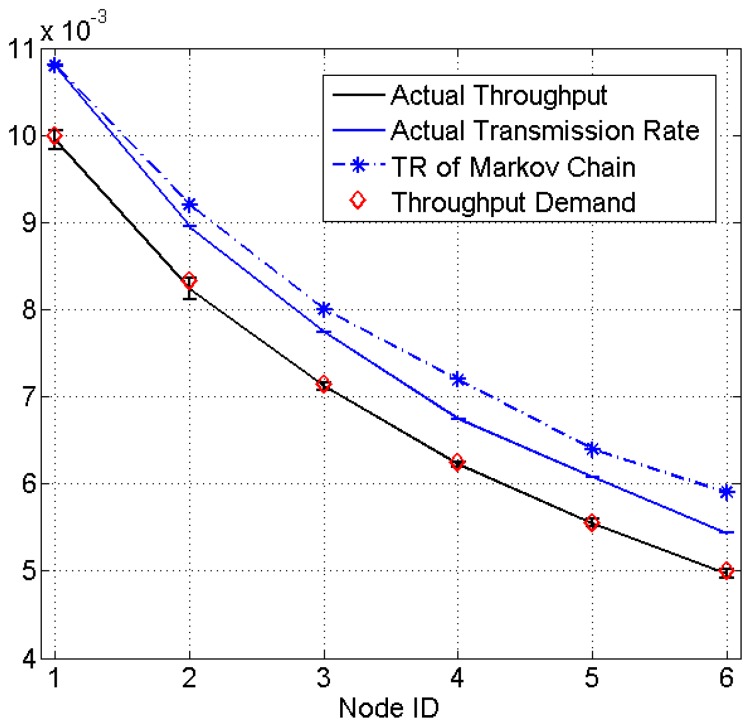
Experiment results of heterogeneous networks (TR: transmission rate).

Figure 7 presents the result for a heterogeneous network consisting of six sensor nodes which are assigned a throughput demand of 1/100, 1/120, 1/140, 1/160, 1/180, 1/200, respectively. The experiment is run for 300 s and repeated 10 times. The actual throughput for each node matches very well with the demands, with an average percentage error of 0.524%.

Table 1 compares the proposed method with the Markov chain-based methods. It is shown that the proposed one is able to satisfy the throughput demand with a smaller error than the one achieved by the Markov chain model in both homogeneous networks and heterogeneous networks. This may be because the Markov chain model relies on certain assumptions (e.g., busy channel probability does not depend on backoff stages), which is prone to slight estimation errors. On the other hand, while the proposed algorithm also makes use of such models, the real-time CCA feedbacks can help to correct the modeling error and lead to a more accurate operating point with respect to the throughput demand. Moreover, due to its distributed nature, the proposed algorithm is easier to implement and requires local channel sensing information only while the Markov chain method is more computationally expensive and requires certain network-wide information. Lastly, the proposed method employs a real-time traffic estimation method and is able to adapt to possible traffic changes while in the case of the Markov chain method, a new model will need to be solved when there is a traffic change.

**Table 1:** Comparisons of the proposed method and the Markov-chain based Methods.

Method		Proposed Method	Markov Chain-Based Methods
Errors	Homogeneous	0.43%	3.2%
	Heterogeneous	0.524%	4.7%
Complexity		easy to implement; able to be stored in a look-up table	computationally intensive; requires solving of multi-dimension Markov chain
Required Information		local information only, e.g., channel sensing result	needs network-wide information, e.g., the number of active nodes
Flexibility		able to adapt traffic changes	requires the traffics to be constant

**Figure 8:**
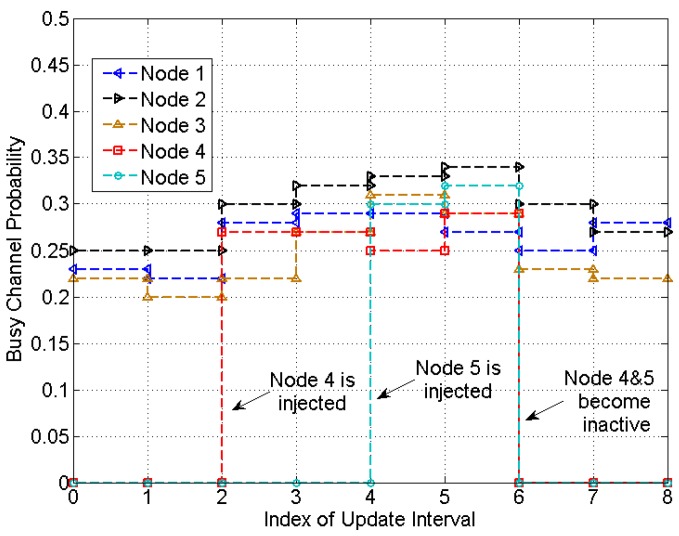
Busy channel probability in a heterogeneous network with changes on the number of nodes.

**Figure 9:**
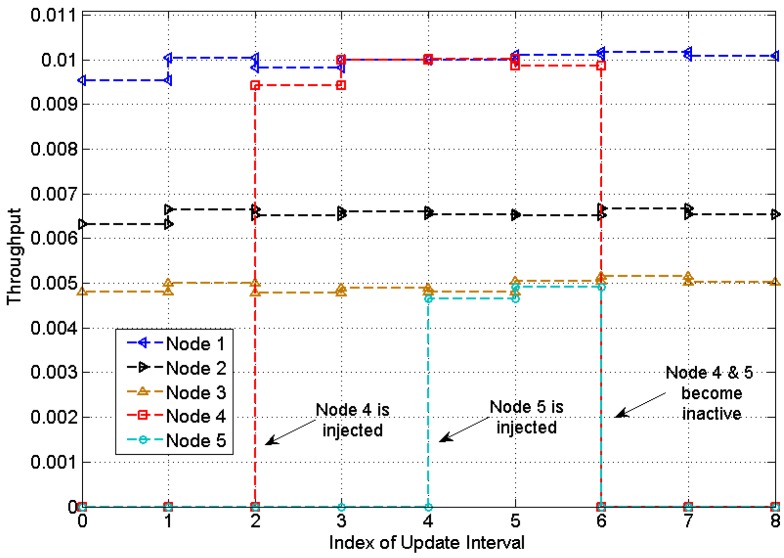
Actual throughput in a heterogeneous network with changes on the number of nodes.

Figure 8 and Figure 9 show the experimental results for a network with changes on the number of nodes. Initially Node 1, Node 2 and Node 3 are in the network with throughput demands of 1/100, 1/150, 1/200, respectively. At the second update, Node 4 is injected with a throughput demand of 1/100, followed by injection of Node 5 with a throughput demand of 1/200 at the 4th update. Node 4 and Node 5 become inactive at the 6th update. The update time is set to be 120 s and the threshold ${\text{β}}_{THR}$ is set to 0.02.

Figure 8 shows the results of busy channel probability (BCP). The BCPs of Node 1–3 changes significantly when Node 4 is injected, with an increment of 0.06, 0.05, 0.02, respectively. According to Section 4, the change of BCP due to the injection of Node 4 is 0.0598 and the maximal fluctuations of the BCPs of the three without any traffic changes are 0.0234, 0.0287 and 0.0331, respectively. Node 3 with the lowest transmission rate suffers most from the noisy measurement of BCP. Nevertheless, as 0.0598 is greater than the maximal fluctuations and all nodes are able to detect the injection of Node 4. However, the detection of Node 5 is difficult as it brings a lower traffic. The BCPs do not change significantly. When Node 4 and Node 5 become inactive, the BCPs of the remaining nodes change significantly, with decrement of 0.02, 0.04 and 0.06.

Despite the fluctuations on BCP, each node is able to adjust the transmission rate to meet the throughput demand. The actual throughput is plotted in Figure 9. It is observed that the average throughput of each node is very close to the demand, with an average percentage error of 0.875% at the optimal point.

## 6. Conclusions

This paper presents a fully distributed rate adjustment algorithm for heterogeneous CSMA/CA networks with time-varying traffics. The algorithm enables each node in the CSMA/CA network to tune its transmission rate to a desired level with respect to a given throughput demand without any central coordination.

The novelty of the proposed algorithm lies in the following three aspects. First, to the best knowledge of the authors, it is the first research work that provides a mathematical characterization of heterogeneous traffics in an IEEE 802.15.4 CSMA/CA network. By exploiting the CCA information, each participating node can accurately determine the level of contention in the network in terms of aggregate transmission rate. Second, with the knowledge of the traffics in the network, each node is able to estimate the packet success rate at equilibrium point and adjust the transmission rate to meet the throughput demand. Compared with existing methods that typically has a prediction error of several percent, the proposed algorithm only yields an average error of 0.43% for homogeneous networks and 0.524% for heterogeneous networks and does not require either complex modeling or central coordination. Third, the algorithm is more flexible in that it is robust against time-varying traffics and can adjust to a new transmission rate should there be any traffic changes.

The proposed algorithm can be used for both homogeneous and heterogeneous CSMA/CA networks. Particularly, it is most suitable for applications with certain throughput requirements. For example, in an intra-vehicular or intra-satellite control system, it is often required that every sensor send a minimum number of measurement packets to the controller to guarantee certain control performance. In this case, the algorithm can be employed to determine the actual transmission rate required. As for practical considerations, the proposed method simply makes use of CCA information and does not require modifications on the CSMA/CA protocol. It is thus easy to implement and computationally inexpensive with the use of a look-up table. Our implementation based on the TinyOS [37,39] platform also shows its compatibility with prevailing protocol stack.

It should be noted that the proposed algorithm can only be applied to IEEE 802.15.4-like CSMA/CA networks. The extension to other forms of CSMA/CA networks (e.g., IEEE 802.11) is possible but requires a different modeling approach, which is beyond the scope of this paper. It should also be noted that the proposed research considered a star-topology and the extension to other topologies is also beyond the scope of this paper.

Future works can be pursued in the following aspects. First, this paper considers each CCA result to be a binary value (*i.e.*, busy/idle). In fact, since each CCA may take up at most two slots and there are four possible outcomes, it is possible to obtain more information on the level of contention by further processing the results. This may also help to propose novel CCA strategies (e.g., the additional carrier sensing method shown in [19]). It is also possible to take into account the imperfect carrier sensing into the modeling [41]. Second, the combination of advanced estimation techniques such as extended Kalman filter and the proposed algorithm may help reduce the error. Third, for a distributed network, there are various forms of local information that can be exploited. For example, the acknowledgement packet (ACK) from the receiver also reflects how busy the network is. Combining the CCA with the ACK can better characterize the network condition. Fourth, in the experiment setup, this paper considers a typical laboratory environment where nodes are randomly placed among cubicles. Future works may consider other typical customized configurations and more extensive experiments can be performed. This may help test scenarios of hotspot as well as bottlenecks and identify potential performance issues in more detail. Lastly, as it is very common that IEEE 802.15.4 devices co-exist with IEEE802.11 (WiFi) networks, it would be practically significant to study the traffic characterization and transmission rate control in the presence of competing networks.
